# Examining the Effects of the AgingPLUS Program on Physical Activity: A Multiple Mediator Model

**DOI:** 10.1093/geroni/igab046.1682

**Published:** 2021-12-17

**Authors:** George Rebok, David Roth, Shang-En (Michelle) Chung, Kaigang Li, Diana Rodriguez, Katherine Thompson, Manfred Diehl, Abigail Nehrkorn-Bailey

**Affiliations:** 1 Johns Hopkins University, Baltimore, Maryland, United States; 2 Colorado State University, Fort Collins, Colorado, United States

## Abstract

This study examined the effect of the AgingPLUS program on anticipated physical activity (PA) and PA engagement, along with the hypothesized mediator roles of self-efficacy (SE) and exercise intention (EI). Data came from 147 participants (Mage = 60.11 years; SD = 8.28 years) of the ongoing trial. Structural equation modeling tested the effects of the intervention, week 4 EI, and week 4 SE on anticipated PA at week 8 and engagement in PA at 6 months. The pathway from week 8 anticipated PA to month 6 PA was also assessed. Results showed that week 4 SE significantly mediated the pathway of intervention condition to week 8 anticipated PA, whereas week 4 EI significantly mediated the pathway from intervention condition to engagement in PA at 6 months. Furthermore, anticipated PA predicted subsequent engagement in PA. Results from these analyses provide preliminary support for the efficacy of the AgingPLUS program.

